# Predicting prolonged work absence due to musculoskeletal disorders: development, validation, and clinical usefulness of prognostic prediction models

**DOI:** 10.1007/s00420-025-02129-8

**Published:** 2025-04-08

**Authors:** Tarjei Rysstad, Margreth Grotle, Adrian C. Traeger, Lene Aasdahl, Ørjan Nesse Vigdal, Fiona Aanesen, Britt Elin Øiestad, Are Hugo Pripp, Gwenllian Wynne-Jones, Kate M. Dunn, Egil A. Fors, Steven J. Linton, Anne Therese Tveter

**Affiliations:** 1 Department of Rehabilitation Science and Health Technology, Faculty of Health Sciences, Oslo Metropolitan University, St. Olavs Plass, P.O. Box 4, 0130 Oslo, Norway; 2 Department of Research and Innovation, Division of Clinical Neuroscience, Oslo University Hospital, Oslo, Norway; 3 Institute for Musculoskeletal Health, The University of Sydney and Sydney Local Health District, Sydney, Australia; 4 School of Public Health, Faculty of Medicine and Health, The University of Sydney, Sydney, Australia; 5 Department of Public Health and Nursing, Faculty of Medicine and Health Sciences, Norwegian University of Science and Technology (NTNU), Trondheim, Norway; 6 Unicare Helsefort Rehabilitation Centre, Rissa, Norway; 7 National Institute of Occupational Health, Majorstuen, Oslo, Norway; 8 Oslo Centre of Biostatistics and Epidemiology, Research Support Services, Oslo University Hospital, Oslo, Norway; 9 Faculty of Health Sciences, Oslo Metropolitan University, Oslo, Norway; 10 School of Medicine, Keele University, Staffordshire, UK; 11 Department of Law, Psychology, and Social Work, Örebro University, Orebro, Sweden; 12 Center for Treatment of Rheumatic and Musculoskeletal Diseases (REMEDY), Diakonhjemmet Hospital, Oslo, Norway

**Keywords:** Prediction model, Prolonged work absence, Musculoskeletal disorders, External validation, Clinical utility, Multivariable logistic regression

## Abstract

**Purpose:**

Given the lack of robust prognostic models for early identification of individuals at risk of work disability, this study aimed to develop and externally validate three models for prolonged work absence among individuals on sick leave due to musculoskeletal disorders.

**Methods:**

We developed three multivariable logistic regression models using data from 934 individuals on sick leave for 4–12 weeks due to musculoskeletal disorders, recruited through the Norwegian Labour and Welfare Administration. The models predicted three outcomes: (1) > 90 consecutive sick days, (2) > 180 consecutive sick days, and (3) any new or increased work assessment allowance or disability pension within 12 months. Each model was externally validated in a separate cohort of participants (8–12 weeks of sick leave) from a different geographical region in Norway. We evaluated model performance using discrimination (*c*-statistic), calibration, and assessed clinical usefulness using decision curve analysis (net benefit). Bootstrapping was used to adjust for overoptimism.

**Results:**

All three models showed good predictive performance in the external validation sample, with *c*-statistics exceeding 0.76. The model predicting > 180 days performed best, demonstrating good calibration and discrimination (*c*-statistic 0.79 (95% CI 0.73–0.85), and providing net benefit across a range of decision thresholds from 0.10 to 0.80.

**Conclusions:**

These models, particularly the one predicting > 180 days, may facilitate secondary prevention strategies and guide future clinical trials. Further validation and refinement are necessary to optimise the models and to test their performance in larger samples.

**Supplementary Information:**

The online version contains supplementary material available at 10.1007/s00420-025-02129-8.

## Introduction

Musculoskeletal disorders are a leading cause of sick leave and disability worldwide, resulting in a significant burden on individuals, employers, and society in terms of lost workdays, healthcare costs, and reduced quality of life (Bevan 2015). Despite only a small proportion of those on sick leave experiencing prolonged sickness absence, they contribute to more than 30% of total days off and 75% of all costs (Henderson et al. 2005). Consequently, current international guidelines for musculoskeletal disorders recommend stratified care using a prognostic prediction model to match the treatment to individuals’ risk profiles (National Guideline Centre (Great Britain) 2016; Foster et al. 2018). However, to effectively implement targeted interventions for reducing sickness absence, accurate and reliable prognostic models are needed to identify individuals at risk of prolonged work absence.

Although various prognostic models exist, few have proved effective in preventing work disability. A recent systematic review concluded that there is weak evidence for any existing prognostic models to accurately predict prolonged sickness absence in individuals with musculoskeletal disorders (Wynne-Jones et al. 2024). Furthermore, this review highlights that few models have undergone external validation, a crucial step toward their clinical implementation (Moons et al. 2012; Steyerberg et al. 2016). Many studies neither adhere to best-practice guidelines for prognostic modelling nor assess clinical utility, such as how well it might guide decisions in clinical settings (Steyerberg et al. 2013; Collins et al. 2015). Notably, decision curve analysis has not yet been used to demonstrate the practical benefits or drawbacks of a prognostic model in the field of work disability.

Another unresolved question is when to identify workers at highest risk. While some evidence suggests that the period between 4 and 12 weeks of sick leave is a critical window for effective intervention (Schultz et al. 2016; Aasdahl and Fimland 2019), several existing models have not examined this timeframe. Early identification is crucial, as the likelihood of returning to work diminishes with increasing sick leave duration (Cancelliere et al. 2016). Developing accurate prognostic models within this timeframe could facilitate early identification and enable stratified care, ensuring that those at highest risk of prolonged sickness absence receive targeted interventions.

To address these gaps, this study aimed to develop and externally validate three prognostic prediction models for prolonged work absence due to musculoskeletal disorders among individuals in the 4–12-week sick leave window. By adhering to established reporting standards and incorporating decision curve analysis to evaluate clinical usefulness, we sought to enhance both the methodological rigor and practical relevance of these models. We developed them within a social insurance context in Norway and validated using an independent regional cohort, thereby strengthening their potential applicability to broader settings.

## Methods

This study was guided by the PROGRESS framework (Steyerberg et al. 2013), and reported in accordance with the Transparent Reporting of a Multivariable Prediction Model for Individual Prognosis or Diagnosis (TRIPOD) statement (Collins et al. 2015) (Online Resource S1 Checklist). The statistical analysis plan was prospectively registered in ClinicalTrials (Registration number: NCT04196634). This study was approved by the Norwegian Centre for Research Data (NSD 861249) and the Regional Committees for Medical and Health Research Ethics in South East Norway (No: 2016/2300), and was conducted in accordance with the Helsinki declaration and the General Data Protection Regulation (GDPR). Written consent was obtained from all participants.

### Study population and data collection

We developed the prognostic prediction models using data from two sources: a prospective cohort study (Tveter et al. 2020) and a multi-arm randomised controlled trial (RCT) (MI-NAV trial) (Aanesen et al. 2022). The cohort study, conducted between November 2018 and February 2019, included 549 workers aged 18–67 years on sick leave for at least 4 weeks due to musculoskeletal disorders, as classified by the International Classification of Primary Care (ICPC-2) (WONCA Classification Committee and Committee 1998). Participants were recruited via the Norwegian Labour and Welfare Service’s (NAV) website, and individuals unemployed or with insufficient Norwegian or English language skills were excluded. The MI-NAV trial, conducted from April 2019 to October 2020, consisted of 514 workers on sick leave for at least 7 weeks due to musculoskeletal disorders, recruited by telephone, excluding individuals with serious health conditions, pregnancy, unemployment, or inadequate language skills. For the prediction models, we used data from the initial 4 to 12 weeks of each participant's sickness absence. Consequently, individuals with sickness absence spells exceeding 12 weeks at the time of data collection were excluded. External validation was conducted using data from a second RCT (Aasdahl et al. 2018), conducted between August 2017 and October 2022 in a different geographical region in Norway. This trial included 865 participants, aged 18–60 years who had been on sick leave for more than 8 weeks. Individuals who were not employed or pregnant were excluded. We further excluded those on sick leave for reasons other than musculoskeletal disorders or sickness absence for more than 12 weeks.

Across these data sources, participants completed a digital questionnaire consisting of validated instruments (Øiestad et al. 2020; Tveter et al. 2020). A small subset of participants was given the option of a paper-based version if digital access was not feasible. These data were linked to the National Sick Leave Registry, providing a 24-month record of sickness absence for each individual, covering a 12-month period before and 12 months after the completion of the baseline questionnaire. This data included information on sick leave benefits and certificates, work assessment allowance (WAA), disability pension (DP), and contracted work hours.

### Outcomes

To operationalise prolonged sickness absence, we defined three outcomes: (1) more than 90 days of work absence during first sick leave spell; (2) more than 180 days of work absence during first sick leave spell; and (3) any new episode or increase in WAA/DP during 1-year follow-up. A *continuous sick leave spell* was measured by counting consecutive calendar days absent from work (including part-time sick leave). This spell ended once the participant returned to at least 80% of their contracted working hours for a minimum of four consecutive weeks. These dichotomous outcomes were consistent across both the development and validation cohorts.

### Model predictors

We developed one multivariable prediction model for each outcome, and selected predictors based on a systematic review of the literature and expert opinions, using sociodemographic and clinical data from patient-reported questionnaires. We used the ‘full model approach’ for model development, meaning all pre-selected predictors were included without exclusion at later stages (Steyerberg 2019). This method is less reliant on data-driven selection and reduces the risk of omitting clinically relevant predictors (Royston et al. 2009; Harrell 2015). Pre-selected predictors were evaluated blinded to the outcome (i.e., exploratory unadjusted analysis was not performed to inform the predictor selection). We followed recent sample size recommendations by Riley et al. (2019, 2020) (Online Resource S1 Supporting Information). For external validation, we adhered to the recommendation of a minimum of 100 outcome events when interpreting the results (Collins et al. 2016). Based on our sample size calculation, we limited the number of predictors to 11 for model development, while accounting for a total of 19 predictor parameters. These predictors, including age, education level, return-to-work expectancy, pain intensity, depression/anxiety, general health, fear avoidance, pain catastrophising, workability, and previous sick leave, have shown robust evidence in predicting sickness absence (Tousignant-Laflamme et al. 2023). All the predictor variables were available in the same form and measurement unit in both datasets, except for the fear-avoidance predictor which differed somewhat between the development and validation samples. Measurement method and variable type of all predictors are presented in Online Resource S1 Table.

### Missing data

Missing data for each prognostic factor is provided in Table 1. No missing data were observed for the three outcomes. As there were hardly any missing data (1.9%) on predictors in the development sample, we conducted complete case analyses for the model development. Out of 336 individuals in the validation sample, 91 had all their predictor data missing. They were therefore excluded from the sample, leaving 244 (72.6%) individuals for the analyses. Of the 244 participants available, 18.4% had missing data on predictor variables which was handled using multiple imputation by chained equations. We created 40 imputed datasets for missing variables that were then combined across all datasets using Rubin’s rule (Marshall et al. 2009) (Online Resource S2 Supporting Information).

**Table 1:** Characteristics of the development and validation samples and the proportion of missing data

	Development sample (*n* = 934)		Validation sample (*n* = 244)	
	Mean (SD) or *n* (%)	Missing data, *n* (%)	Mean (SD) or *n* (%)	Missing data, *n* (%)
Sociodemographic factors				
Age, years	47.9 (10.5)	0	45.3 (9.8)	4
Gender, women	540 (57.8%)	0	140 (57.4%)	0
Education level		0		1 (0.4)
Primary/secondary/vocational school	575 (61.5%)		129 (52.9%)	
College/university	358 (38.3%)		114 (46.7%)	
Work characteristics				
Sick leave previous year, days, median (IQR)	35.0 (26.4–49.6)	0	73.1 (58.9–91.4)	0
Granted DP prior to baseline	59 (6.3%)	0	11 (4.5%)	0
Workability, 0-10^b^	3.2 (2.7)	4 (0.4)	3.4 (2.7)	5 (2.0)
RTW expectancy, 0-10^b^	7.0 (2.9)	0	7.3 (3.2)	15 (6.1)
Symptom and psychological characteristics				
Pain last week, 0-10^a^	5.9 (2.1)	0	5.6 (2.1)	10 (4.1)
Fear avoidance, yes	505 (54.1%)	0	32 (13.1%)	0
Health, 0-100^b^	52.2 (20.1)	7 (0.8)	52.5 (21.5)	28 (11.5)
Anxiety/depression		7 (0.8)		12 (4.9)
No/little	713 (76.3%)		203 (83.2%)	
Moderate	159 (17.0%)		22 (9.0%)	
Severe/extreme	55 (5.9%)		7 (2.9%)	
Outcomes				
> 90 SA days	416 (44.5%)	0	124 (50.8%)	0
> 180 SA days	161 (17.2%)	0	70 (28.7%)	0
New/increase in DP/WAA	101 (10.8%)	0	43 (17.6%)	0

*DP* disability pension, *IQR* interquartile range, *SA* sickness absence, *WAA* work assessment allowance

^a^Higher score is worse

^b^Higher score is better

### Statistical analysis

#### Sample relatedness

To assess the case-mix and relatedness between the development and validation samples, we followed the framework by Debray et al. (2015). Specifically, we created a ‘membership model’ using logistic regression to predict the probability of an individual being a member of the development sample rather than the validation sample. Independent variables in this membership model were the outcomes, gender, and all predictors from the prediction models. We used *c*-statistic to quantify the case-mix between the samples, where low values (close to 0.5) indicate similar case-mix and related samples.

#### Model development

We fitted three multivariable logistic regression models using the pre-selected predictors, without additional statistical selection processes. To account for data sourced from RCTs, intervention above usual care (presence or absence) was included as a predictor to mitigate bias from trial effects (Pajouheshnia et al. 2019). Continuous predictors were kept as continuous, and non-linearity and functional form was assessed using fractional polynomials (Sauerbrei and Royston 1999). We found that all the continuous variables were better modelled using a linear function. Model performance was evaluated using both apparent and optimism-corrected measures. Internal validation involved bootstrap resampling (500 samples) to adjust for overfitting, leading to shrunken regression coefficients and performance measures (Harrell Jr et al. 1996). Model performance was reported across three dimensions: overall model performance measured by Nagelkerke’s *R*^2^, discrimination, and calibration. Nagelkerke’s *R*^2^ estimates the proportion of variance explained by the model, ranging from 0–1 (1 = perfect). Model discrimination (i.e., the ability to distinguish between those with positive and negative outcomes) was assessed using *c*-statistic, equivalent to the area under the curve (AUC) of the receiver operator characteristic (ROC), with values ranging from 0.5 (no better than chance) to 1 (perfect discrimination). Model calibration (i.e., how well predicted probabilities agree with observed events) was assessed using calibration plot, calibration-in-the-large, calibration slope, and the ratio of the expected and observed event probabilities (E/O ratio). Calibration plot shows the observed and predicted probabilities of incident long-term sick leave by deciles of predicted risk, and we applied a locally weighted scatterplot smoother (lowess) curve to show calibration across the entire range of predicted probabilities. Calibration-in-the-large (intercept) is the difference between the mean observed risk and mean predicted risk and is a measure of systematic overprediction or underprediction, with an ideal value of 0. Calibration slope is a measure of agreement of observed and predicted risk across the whole range of predicted values, with a slope of 1 indicating perfect calibration; slope > 1 indicates that the risk predictions are too narrow, whereas slope < 1 indicates that the risk predictions are too extreme. Lastly, we assessed the E/O ratio to summarise the overall calibration, in which perfect calibration is represented by a value of 1.

#### External validation

We externally validated the optimism-adjusted prediction models by assessing the performance of the models when applied to the external validation sample. Model coefficients were averaged over the imputed datasets using Rubin’s rule (Steyerberg 2019). Performance criteria comprised overall performance, discrimination, and calibration as described above.

#### Clinical usefulness

We used decision curve analysis to evaluate and compare the clinical usefulness of our models in the external validation sample. Decision curve analysis is a plot of the net benefit (analogous to profit) against the threshold probabilities of the prognostic model. Net benefit of a prognostic model is the difference between the proportion of true positives and the proportion of false positives weighted by the odds of the selected threshold for the outcome (Vickers and Elkin 2006; Vickers et al. 2019). Hence, to assess the clinical usefulness, we plotted decision curves for the models, and for two default strategies: ‘treating all’ and ‘treating none’. The strategy with the highest net benefit at any given risk threshold is considered to have the most clinical value (Vickers et al. 2019).

All statistical analyses were conducted using Stata version 17.0 (StataCorp, College Station, TX, USA), using the bsvalidation, mfpmi, pmcalplot, and dca packages. All analyses were supervised by a biostatistician.

### Sensitivity analyses

To evaluate the impact of multiple imputation, we conducted complete case analyses on our external validation procedure. To assess the predictive ability of the models in a broader sample, we performed sensitivity analyses on all participants in the external validation cohort who were on sick leave between 4 and 12 weeks at baseline irrespective of diagnoses. Similar analyses were conducted including those with musculoskeletal disorders and mental disorders.

## Results

The development sample included 934 individuals (mean age 47.9 years, 57.8% women), while the external validation sample included 244 individuals (mean age 45.3 years, 57.4% women), as detailed in Online Resource S1 Fig. We observed higher event rates in the external validation sample for all outcomes. In the development sample, the event rates for the > 90 days, > 180 days, and DP/WAA outcomes were 44.5%, 17.2%, and 10.8%, respectively, compared to 50.8%, 28.7%, and 17.6% in the external validation sample.

Differences were observed between the development and validation samples, as reported in Table 1. Of note, a higher proportion of participants in the external validation sample had higher education (46.7% vs. 38.3%), and more sick leave the previous year (median days, 73.1 vs. 35.0), but fewer reported moderate (9.0% vs. 17.0%) and severe/extreme (2.9% vs. 5.9%) anxiety/depression symptoms, and fewer reported fear avoidance behaviour (13.1% vs. 54.1%). The *c*-statistic for the membership model comparing the relatedness of the respective samples was 0.91 (95% confidence interval 0.89 to 0.93), indicating highly discordant case-mix.

### Model development

All final prediction models are presented in Table 2. Table 3 presents apparent and internal validation performance measures of our prediction models. After bootstrap adjustment for optimism, all three models were able to discriminate between those with and without the outcomes with a *c*-statistic range from 0.74 to 0.82, with reassuringly narrow confidence intervals. Bootstrap shrinkage factors ranging from 0.89 to 0.93 were applied to adjust the coefficients of the final models (Table 2).

**Table 2:** Final prediction models with predictors, coefficients, 95% confidence intervals, and odds ratio (OR) adjusted by bootstrap shrinkage

Predictors in the models, range	> 90 days		> 180 days		DP/WAA	
	Coefficient	OR (95% CI)	Coefficient	OR (95% CI)	Coefficient	OR (95% CI)
Age, years	0.001	1.00 (0.99; 1.01)	– 0.006	0.99 (0.98; 1.01)	0.017	1.017 (0.99; 1.04)
Educational level, 0–1	0.127	1.14 (0.86; 1.50)	0.306	1.36 (0.93; 1.99)	0.205	1.23 (0.78; 1.95)
Pain intensity, 0–10	0.048	1.05 (0.98; 1.13)	0.082	1.09 (0.99; 1.19)	0.053	1.05 (0.94; 1.19)
RTW expectancy, 0–10	– 0.195	0.82 (0.78; 0.87)	– 0.206	0.81 (0.76; 0.87)	– 0.181	0.83 (0.78; 0.90)
Sick leave days previous year, days	0.006	1.01 (1.00; 1.01)	0.128	1.01 (1.01; 1.02)	0.016	1.02 (1.01; 1.02)
General health, 0–100	– 0.008	0.99 (0.98; 0.99)	0.004	1.00 (0.99; 1.01)	– 0.001	0.99 (0.99; 1.01)
Depression/anxiety						
None/little		1		1		1
Moderate	– 0.214	0.81 (0.56; 1.17)	– 0.434	0.65 (0.40; 1.06)	0.141	1.15 (0.66; 2.00)
Very/extreme	– 0.257	0.77 (0.43; 1.38)	– 0.052	0.95 (0.49; 1.84)	0.295	1.34 (0.63; 2.88)
Workability, 0–10	– 0.160	0.85 (0.80; 0.90)	– 0.215	0.81 (0.74; 0.88)	– 1.044	0.90 (0.81; 0.99)
Fear avoidance, 0–1	– 0.104	0.90 (0.68; 1.19)	– 0.040	0.96 (0.66; 1.39)	– 0.102	0.90 (0.57; 1.42)
Disability pension, 0–1	– 0.023	0.98 (0.97; 0.99)	– 0.064	0.94 (0.90; 0.97)	0.009	1.01 (0.99; 1.02)
Intervention, 0–1	0.078	1.08 (0.82; 1.43)	– 0.276	0.76 (0.52; 1.11)	– 1.714	0.18 (0.09; 0.35)
Intercept	1.540	4.67 (4.05;5.38)	– 0.568	0.57 (0.46; 0.68)	– 2.410	0.09 (0.07; 0.11)

All binary variables (0–1) are coded 1 when present and 0 when absent

RTW, return to work

**Table 3:** Predictive performance of the prognostic models

Performance measure	Development sample (*n* = 934)		Validation sample (*n* = 244)
	Apparent performance (95% CI)	Optimism adjusted (Bootstrap 95% CI)*	External validation (95% CI)
Outcome 1: > 90 days absence, *n* events (%)	416 (44.5)		124 (50.8)
*c*-statistic	0.75 (0.72;0.78)	0.74 (0.71;0.77)	0.78 (0.72;0.84)
Calibration slope	–	0.93 (0.77;1.09)	1.15 (0.82;1.48)
Calibration-in-the-large	–	– 0.01 ( – 0.15;0.14)	0.17 ( – 0.11;0.45)
Expected: Observed ratio	–	1.00 (0.94;1.07)	0.93
Nagelkerke’s *R*^2^	0.24	0.24	0.31
Outcome 2: > 180 days absence,* n* events (%)	161 (17.2)		70 (28.7)
*c*-statistic	0.80 (0.76;0.84)	0.78 (0.74;0.82)	0.79 (0.73;0.85)
Calibration slope	–	0.92 (0.76;1.11)	0.95 (0.66;1.23)
Calibration-in-the-large	–	0.00 ( – 0.18;0.19)	0.14 ( – 0.18;0.46)
Expected: Observed ratio	–	1.02 (0.89;1.15)	0.93
Nagelkerke’s *R*^2^	0.28	0.28	0.30
Outcome 3: DP/WAA, *n* events (%)	101 (10.8)		43 (17.6)
*c*-statistic	0.84 (0.81;0.88)	0.82 (0.78;0.86)	0.77 (0.69;0.85)
Calibration slope	–	0.89 (0.73;1.08)	0.92 (0.58;1.26)
Calibration-in-the-large	–	0.00 ( – 0.25;0.27)	– 0.25 ( – 0.61;0.12)
Expected: Observed ratio	–	1.00 (0.83;1.18)	1.18
Nagelkerke’s *R*^2^	0.30	0.30	0.21

*CI* confidence interval, *DP* disability pension, *WAA* work assessment allowance

*Refers to performance estimated after bootstrap optimism adjustment (500 replications). The optimism adjusted calibration slope is equivalent to the bootstrap uniform shrinkage factor for each model

### External validation

**Fig. 1:**
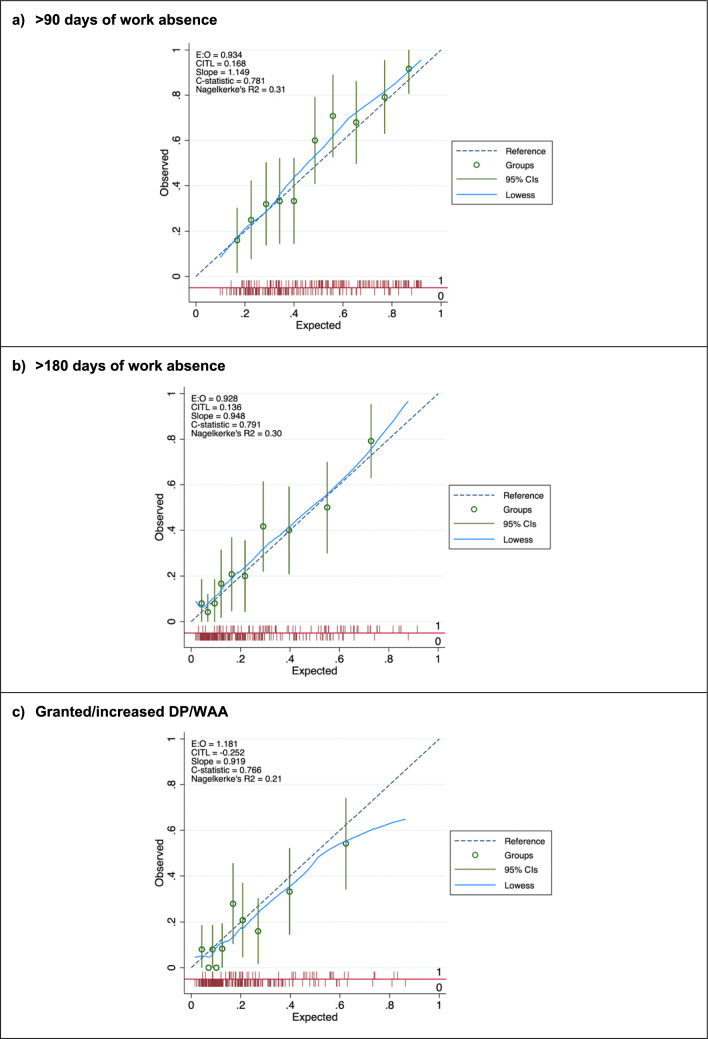
Calibration plots for the prognostic models when applied to the external validation sample. The smoothed (Lowess) line in light blue shows the agreement between predicted and observed probabilities of prolonged sickness absence outcomes. The dashed diagonal line indicates perfect calibration. The circled points represent observed proportion of prolonged sickness absence in decile groups of predicted risk, with vertical lines representing 95% confidence intervals. The spike plot on each *x*-axis summarises the density of participants with outcome (1) and without outcome (0)

Among the three models, the > 180-day model stood out, achieving the highest predictive performance in the external validation. As detailed in Table 3, both the > 90 and > 180 days models showed good overall performance with Nagelkerke’s *R*^2^ ranging from 0.31 to 0.33, whereas the DP/WAA model had a modest *R*^2^ of 0.21. The *c*-statistic was 0.78 (95% CI 0.72–0.84) for the > 90 days model, 0.79 (95% CI 0.73–0.85) for the > 180 days model, and 0.77 (95% CI 0.69–0.85) for the DP/WAA model. Overall, the calibration plots indicated good agreement between predicted and observed risks for both the > 90 days and > 180 days models, whereas for the DP/WAA model, the predictions were overestimated in the three higher-risk groups (Fig. 1). The > 180 days model demonstrated very good calibration, with a calibration slope slightly below 1 and a calibration-in-the-large somewhat above 0. Compared with the development sample, the > 180 days model’s *c*-statistic increased slightly from 0.78 (95% CI 0.74–0.82) to 0.79 (95% CI 0.73–0.85), and Nagelkerke’s R^2^ increased from 0.28 to 0.30, indicating a slight improvement in model performance in the external cohort. Further details of this model and its calculation can be found in Online Resource S3 Supporting Information. The distribution of predicted probabilities for individuals with and without outcomes is presented for each model in Online Resource S2 Fig.

### Clinical usefulness

**Fig. 2:**
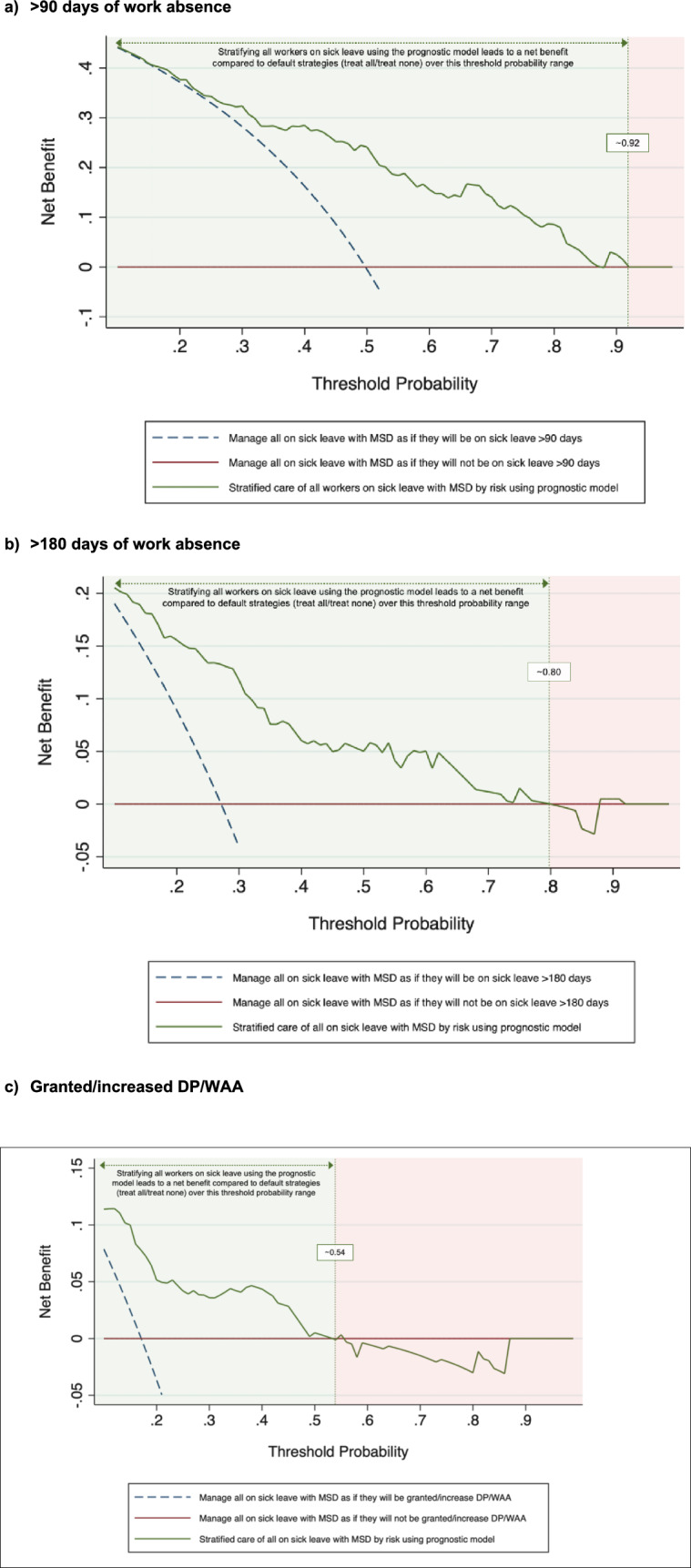
External validation decision curve analysis for the net benefit of the prognostic models with default strategies (treat all and treat none). Net benefit represents the treatment threshold weighted sum of true- minus false-positive classifications for each strategy plotted against an entire range of treatment thresholds. At a risk threshold of 0.5, 1 true positive would be balanced by 1 false positive. Red line: no individuals are treated, net-benefit is zero (no true-positive and no false-positive classifications); blue dashed line: all individuals are treated; green line: all individuals are treated based on stratified care using the prognostic models. Between thresholds that are highlighted in light green, treating individuals based on our prognostic models is superior to treating all or treating non

Figure 2 illustrates the decision curve analyses, showing net benefit of the prediction models compared with two default strategies (treat all or treat none) for decisions about management of individuals on sick leave due to musculoskeletal disorders. The decision to use stratified care based on the prediction models had greater net benefit (i.e., clinical utility) than the default strategies in a wide range of meaningful predicted probabilities, with the model predicting > 180 days showing the greatest net benefit across the widest probability thresholds. For this model, the net benefit exceeded both default strategies at risk thresholds between 10 and 80% (Fig. 2b). The > 90 days model performed well across a broad range of thresholds, with net benefit being comparable to the treat-all strategy up to 29% and superior between 40 and 92% (Fig. 2a). The DP/WAA model, while still providing some clinical value, had a more limited range of net benefit (10–50%) and tended to overestimate risk in higher probability groups (Fig. 2c). Because the > 180-day model demonstrated consistently higher net benefit at most thresholds, it indicates potential clinical value for guiding targeted interventions in individuals at risk of prolonged sick leave.

### Sensitivity analyses

When externally validating the prediction models in complete case data (n = 199), all three models showed slightly reduced performance compared to the imputation sample (Online Resource S3 Fig). Specifically, the *c*-statistics declined from 0.78 to 0.75 for the > 90 days model, from 0.79 to 0.75 for the > 180 days model, and from 0.77 to 0.74 for the DP/WAA model. While discrimination remained acceptable, the calibration slopes also changed slightly (from 0.93 to 0.86 for > 90 days, from 0.92 to 0.87 for > 180 days, and from 0.89 to 0.82 for DP/WAA). These findings suggest that missing data imputation had a modest impact on model performance, with slightly better calibration and discrimination observed in the full dataset. We also performed sensitivity analysis by testing the models’ predictive performance when including all individuals in the external validation sample (*n* = 600), regardless of diagnosis. The models’ discriminatory ability was somewhat reduced (*c*-statistics 0.74–0.75), while calibration was significantly worse, although the > 90 model exhibited an excellent calibration slope of 0.996 (Online Resource S4 Fig). Somewhat similar results to this were obtained when applying the models to individuals on sick leave due to musculoskeletal disorders or mental disorders (*n* = 418) (Online Resource S5 Fig).

## Discussion

We developed and validated three models to predict prolonged work absence outcomes, using 11 well-defined and easily measurable predictors in a social insurance context. These models demonstrated acceptable accuracy in an external validation sample. Particularly, the > 180 days model showed a substantial net benefit in decision curve analysis, emphasising its practical value in managing musculoskeletal disorder-related sick leave.

Our study identified significant differences in event rates and participant characteristics between the development and external validation samples. The *c*-statistic of 0.91 for the ‘membership model’ suggests that our external validation assessed the geographic transportability of the models (Debray et al. 2015). Despite the known challenges in performance across new settings, particularly calibration is more vulnerable to geographic transportability (Van Calster et al. 2019; Gulati et al. 2022), our models maintained satisfactory performance in external validation, indicating their generalisability. They demonstrated good discriminatory performance (*c*-statistics > 0.77), and this increased for both the > 90 and > 180 days models, indicating little overfitting in the development (Van Calster et al. 2019). While their calibration performance was overall good, their predictions were slightly lower in the external validation (calibration-in-the-large of 0.17 and 0.14, respectively), likely due to the higher event rate in this cohort. However, the > 180 days model had an excellent calibration slope of 0.95, affirming its promising transportability. Conversely, the DP/WAA model faced challenges, indicated by a 9–10% lower *R*^2^, reduced discrimination, and a tendency to overestimate risk predictions (intercept -0.25), especially in the higher-risk groups. This overestimation could potentially lead to overtreatment (Van Calster et al. 2019). Although updating the DP/WAA model could address this issue, we believe the small sample size in higher-risk groups is a primary concern. Therefore, we have opted not to update the model’s slope and intercept as further validation with a larger sample size is needed.

The robust external validity of our models can be attributed to several key design decisions. We adopted a full model approach, informed by previous studies, literature reviews, and clinical insights, to mitigate risks of bias and overfitting. Bootstrapping techniques were employed to ensure model stability, as evidenced by the small shrinkage factors for the > 90 and > 180 days models. We also maintained model parsimony by aligning the number of predictors with our sample size calculation and avoided overcomplexity by modelling continuous variables linearly. This is an important step to prevent model instability, as overly complex models often perform poorly in external validation in similar-sized samples (Steyerberg 2019; Van Calster et al. 2019).

While numerous studies have focused on predicting the risk of long-term sick leave, our research specifically addresses prognostic models for individuals already on sick leave due to musculoskeletal disorders. Instruments like the Örebro Musculoskeletal Pain Screening Questionnaire (Linton et al. 2011) and SIMBO (Streibelt and Bethge 2015) have demonstrated good discriminatory ability but were not developed using multivariable regression. This makes a direct comparison with our models somewhat challenging. A more directly comparable study is that of Ropponen and colleagues (2020), which also employed regression analysis for model development in a similar context. Despite their larger sample size, their model exhibited weaker discriminatory abilities, with *c*-statistics of 0.64 and 0.69 for predicting > 90 and > 180 days of prolonged sick leave, respectively. Additionally, the lack of reported calibration and explained variance (*R*^2^) in their study limits a full performance comparison.

To our knowledge, our study is the first to utilise decision curve analysis to assess the clinical utility of prediction models for prolonged work absence. Both > 90 and > 180 days models performed almost similarly well, except the > 180 model showed higher net benefit at lower risk scores. When the probability threshold is below 0.10 or above 0.80, the > 180 days model does not offer any net benefit over standard treat-all strategies. Despite this, both models outperform the treat-all approach across a wide range of clinically relevant thresholds. The choice of threshold should be tailored to individual and clinical preferences, as well as the context of the social insurance system. The DP/WAA model, however, exhibits net benefits in a more limited threshold range due to miscalibration.

### Strengths and limitations

Our study has several strengths. To our knowledge, this is the first study to develop and validate prediction models for prolonged work absence in line with recent methodological guidelines like the PROGRESS framework (Steyerberg et al. 2013) and TRIPOD (Moons et al. 2015). We followed a pre-established statistical analysis plan, employed a prospective design, and determined sample size based on current recommendations, enhancing methodological rigor, transparency, and reducing biases (Riley et al. 2020). We selected predictors for their relevance and ease of collection, facilitating implementation in various settings, and accounted for intervention effects in our validation process, addressing a common issue in prediction studies (Pajouheshnia et al. 2019). However, there are limitations. The small size of the external validation cohort limits the precision of our estimates and calls for caution in interpretation. This was primarily due to complete missing predictor data, reducing our sample from 334 to 244 participants. We mitigated the impact of partial missing data using multiple imputation techniques (Moons et al. 2015). Nevertheless, a larger sample is required for more accurate validation, recognising that prediction model validation is a continuous process (Van Calster et al. 2023). To enable this, we have provided the underlying model coefficients. The variation in measuring the fear-avoidance predictor between samples is another limitation, potentially affecting model performance (Luijken et al. 2019). Despite this difference, all other predictors and outcomes were consistently measured. The limited sample size also restricted our ability to assess model performance with additional predictors, a key aspect of model updating. Future research should investigate whether integrating new predictors improves predictive accuracy and clinical utility (Nieboer et al. 2016). Furthermore, it is also important to consider case-mix comparison in further external validations and recalibration in settings with varied case-mix, especially if poor calibration is observed (Moons et al. 2015; Steyerberg 2019).

### Practical implications

Our models, designed for the initial 4 to 12 weeks of sick leave due to musculoskeletal disorders, provide individual risk probabilities, offering valuable information for individuals on sick leave, clinicians, and caseworkers. These models also include self-reported modifiable predictors, enabling personalised tailoring of intervention strategies based on each individual’s risk profile. Moreover, the data gathered from these models may enhance clinicians' understanding of prognosis and factors influencing work absence. While these models mark progress in personalised management for sick leave, further validation in diverse settings and determining optimal interventions for high-risk cases are necessary future steps.

In summary, we have developed and validated three prediction models, and the > 180 days model in particular can predict prolonged sickness absence in workers on sick leave due to a musculoskeletal diagnosis with sufficient accuracy. External validation showed that these prediction models are transportable to the same target population but with a different case-mix. These prediction models use 11 easily measured predictors and demonstrated clinical utility over a broad range of clinically relevant thresholds. These models can help reduce healthcare and societal costs by avoiding unnecessary interventions and focusing resources on those who need them the most. Additional external validation and implementation studies are required before the models can be implemented, to assess whether their predictions can improve outcomes in various settings.

## Supplementary Information

Below is the link to the electronic supplementary material.

Supplementary file1 (DOCX 3143 KB)

## Data Availability

Anonymised individual participant data, including the data dictionary, will be available upon reasonable request from January 2023 to December 2028. Researchers should submit a methodologically sound proposal approved by an ethics committee and the MI-NAV study’s scientific board.
