# Supplementary material for: Predicting prolonged work absence due to musculoskeletal disorders: development, validation, and clinical usefulness of prognostic prediction models
Source: Int Arch Occup Environ Health. 2025 Apr 8;98(4-5):385–97. doi: 10.1007/s00420-025-02129-8 (PMC12238208; doi:10.1007/s00420-025-02129-8)
Supplement: Supplementary file 1 — Supplementary file1 (DOCX 3143 KB) [file 420_2025_2129_MOESM1_ESM.docx]

Supplementary information

**Table of contents**

[S1 TRIPOD Checklist. 1](#_Toc182560786)

[﻿﻿S1 Figure. Participant flow diagram. 4](#_Toc182560787)

[S2 Figure. Density of events and non-events across predicted probabilities. 5](#_Toc182560788)

[S3 Figure 6](#_Toc182560789)

[S4 Figure 7](#_Toc182560790)

[S5 Figure 8](#_Toc182560791)

[S1 Supporting Information: Sample size calculation 10](#_Toc182560792)

[S2 Supporting Information: Multiple imputation 11](#_Toc182560793)

[S3 Supporting Information: Predicted Risk Calculation for >180 Days of Prolonged Work Absence with Two Examples 14](#_Toc182560794)

[References 15](#_Toc182560795)

﻿

S1 TRIPOD Checklist. Completed TRIPOD checklist.

| **Section/Topic** | **Item** |  | **Checklist Item** | **Page** |
| --- | --- | --- | --- | --- |
| **Title and abstract** | | | | |
| Title | 1 | D;V | Identify the study as developing and/or validating a multivariable prediction model, the target population, and the outcome to be predicted. | 1 |
| Abstract | 2 | D;V | Provide a summary of objectives, study design, setting, participants, sample size, predictors, outcome, statistical analysis, results, and conclusions. | 5 |
| **Introduction** | | | | |
| Background and objectives | 3a | D;V | Explain the medical context (including whether diagnostic or prognostic) and rationale for developing or validating the multivariable prediction model, including references to existing models. | Introd, para 1-2 |
|  | 3b | D;V | Specify the objectives, including whether the study describes the development or validation of the model or both. | Introd, para 3 |
| **Methods** | | | | |
| Source of data | 4a | D;V | Describe the study design or source of data (e.g., randomized trial, cohort, or registry data), separately for the development and validation data sets, if applicable. | Methods, para 2-3 |
|  | 4b | D;V | Specify the key study dates, including start of accrual; end of accrual; and, if applicable, end of follow-up. | Methods, para 2 |
| Participants | 5a | D;V | Specify key elements of the study setting (e.g., primary care, secondary care, general population) including number and location of centres. | Methods, para 2-3 |
|  | 5b | D;V | Describe eligibility criteria for participants. | Methods, para 2-3 |
|  | 5c | D;V | Give details of treatments received, if relevant. | Referred to original papers, Methods, para 2-3 |
| Outcome | 6a | D;V | Clearly define the outcome that is predicted by the prediction model, including how and when assessed. | Methods, para 6 |
|  | 6b | D;V | Report any actions to blind assessment of the outcome to be predicted. | n.r. |
| Predictors | 7a | D;V | Clearly define all predictors used in developing the multivariable prediction model, including how and when they were measured. | S1 Table |
|  | 7b | D;V | Report any actions to blind assessment of predictors for the outcome and other predictors. | Methods, para 6 |
| Sample size | 8 | D;V | Explain how the study size was arrived at. | Methods, para 6 & S1 Supporting Information |
| Missing data | 9 | D;V | Describe how missing data were handled (e.g., complete-case analysis, single imputation, multiple imputation) with details of any imputation method. | Methods, para 7 & S2 Supporting Information |
| Statistical analysis methods | 10a | D | Describe how predictors were handled in the analyses. | Methods, para 6 & 10 |
|  | 10b | D | Specify type of model, all model-building procedures (including any predictor selection), and method for internal validation. | Methods, para 10 |
|  | 10c | V | For validation, describe how the predictions were calculated. | Methods, para 10 & 11 & Table 2 |
|  | 10d | D;V | Specify all measures used to assess model performance and, if relevant, to compare multiple models. | Methods, para 12 |
|  | 10e | V | Describe any model updating (e.g., recalibration) arising from the validation, if done. | n.r. |
| Risk groups | 11 | D;V | Provide details on how risk groups were created, if done. | n.r. |
| Development vs. validation | 12 | V | For validation, identify any differences from the development data in setting, eligibility criteria, outcome, and predictors. | Methods, para 2-3 |
| **Results** | | | | |
| Participants | 13a | D;V | Describe the flow of participants through the study, including the number of participants with and without the outcome and, if applicable, a summary of the follow-up time. A diagram may be helpful. | Results, para 1 & S1 Figure |
|  | 13b | D;V | Describe the characteristics of the participants (basic demographics, clinical features, available predictors), including the number of participants with missing data for predictors and outcome. | Results, para 2 & Table 1 |
|  | 13c | V | For validation, show a comparison with the development data of the distribution of important variables (demographics, predictors and outcome). | Results, para 2 & Table 1 |
| Model development | 14a | D | Specify the number of participants and outcome events in each analysis. | Results, para 1 & Table 1 |
|  | 14b | D | If done, report the unadjusted association between each candidate predictor and outcome. | Not performed |
| Model specification | 15a | D | Present the full prediction model to allow predictions for individuals (i.e., all regression coefficients, and model intercept or baseline survival at a given time point). | Table 2 |
|  | 15b | D | Explain how to use the prediction model. | Box 1 |
| Model performance | 16 | D;V | Report performance measures (with CIs) for the prediction model. | Table 3 |
| Model-updating | 17 | V | If done, report the results from any model updating (i.e., model specification, model performance). | n.r. |
| **Discussion** | | | | |
| Limitations | 18 | D;V | Discuss any limitations of the study (such as nonrepresentative sample, few events per predictor, missing data). | Discussion, para 6 |
| Interpretation | 19a | V | For validation, discuss the results with reference to performance in the development data, and any other validation data. | Discussion, para 2 |
|  | 19b | D;V | Give an overall interpretation of the results, considering objectives, limitations, results from similar studies, and other relevant evidence. | Discussion, para 1-5 |
| Implications | 20 | D;V | Discuss the potential clinical use of the model and implications for future research. | Discussion, para 7 |
| **Other information** | | | | |
| Supplementary information | 21 | D;V | Provide information about the availability of supplementary resources, such as study protocol, Web calculator, and data sets. | Methods, para 1 |
| Funding | 22 | D;V | Give the source of funding and the role of the funders for the present study. | Funding |

﻿S1 Table. Summary of selected prognostic factors for model development with definition, variable type, unit/categories, measurement method, number of predictor parameters, and evidence.

| Prognostic factor | Definition | Variable type | Unit/categories | Measurement method | No. of predictor parameters |
| --- | --- | --- | --- | --- | --- |
| Expectation of RTW | Expectation of probability of returning to work within 3 months | Continuous | 0-10, 10 = best | Self-reported using item from ÖMPSQ-SF [1] | 1 |
| Pain intensity | Pain intensity last week | Continuous | 0-10, 10 = worst | Self-reported | 1 |
| Depression/  anxiety | Self-reported depression or anxiety | Categorical | Recategorized from 5 to 3 categories | Self-reported using item from EQ5D [2] | 3 |
| Education level | Completed education | Binary | 0 ‘Lower education: primary/secondary/ vocational school’ 1 ‘Higher education: College/University. Recategorized from 4 to 2 categories | Self-reported | 1 |
| Age | Age | Continuous | Years | Self-reported | 1 |
| Previous sick leave | Days of sick leave previous year | Continuous | Days | Register data | 1 |
| Disability pension status | Receiving work assessment allowance or disability pension at starting-point | Binary | 0 ‘No’ 1 ‘Yes’ | Register data | 1 |
| General health | Self-perceived general health | Continuous | 0-100 | Self-reported using VAS from EQ5D [2] | 1 |
| Fear-avoidance | Fear that movement or activity will worsen the injury | Binary | 0 ‘No’ 1 ‘Yes’ | Self-reported. Development sample: “Do you worry that physical activity could make your condition worse?” using item from Keele STarT MSK tool [3,4]  Validation sample:  “Do you think it is best to stay still so that the pain does not get worse?” | 1 |
| Workability (job performance) | Self-reported current ability to work compared to at its best | Continuous | 0-10, 10=best | Self-reported using item from Work Ability Index [5] | 1 |
| Intervention | Participant in a trial arm with an effective intervention | Binary | 0 ‘No’ 1 ‘Yes’ |  | 1 |
|  |  |  |  |  | Total 13 |

﻿﻿S1 Figure. Participant flow diagram.


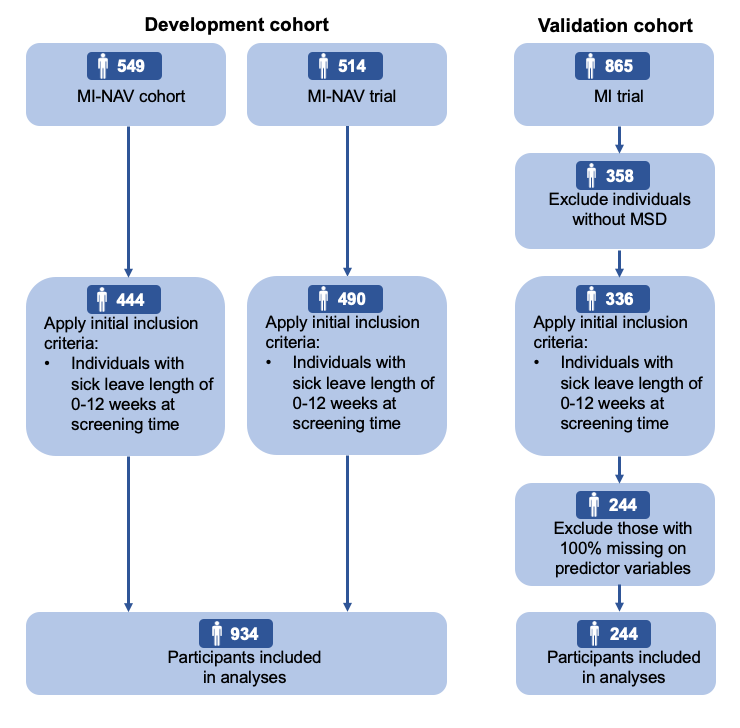


S2 Figure. Density of events and non-events across predicted probabilities.

| >90 days model  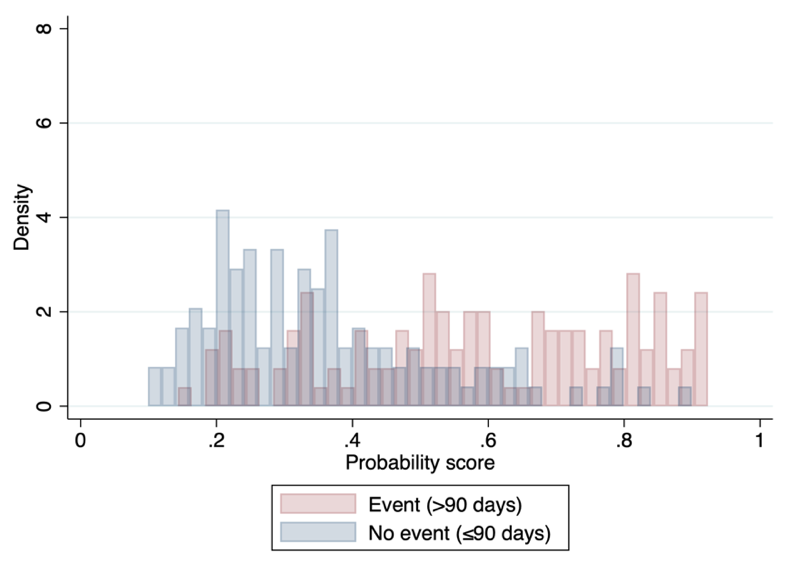 |
| --- |
| >180 days model  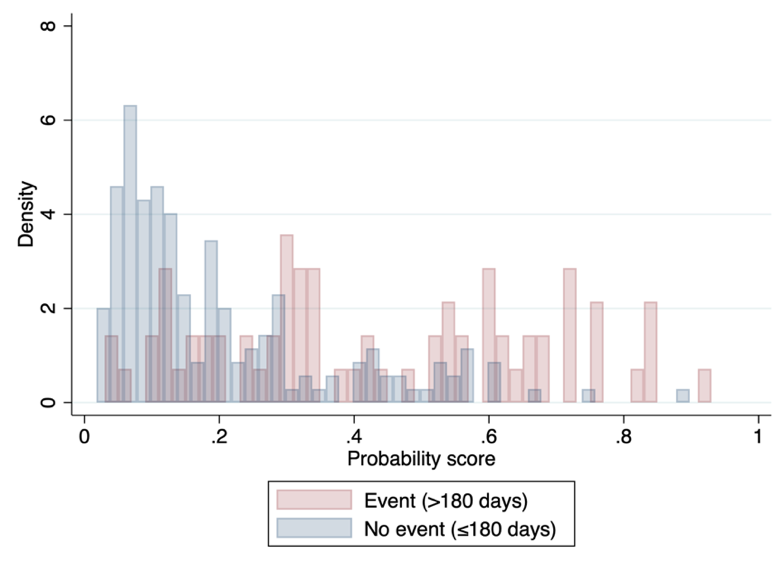 |
| WAA/DP model  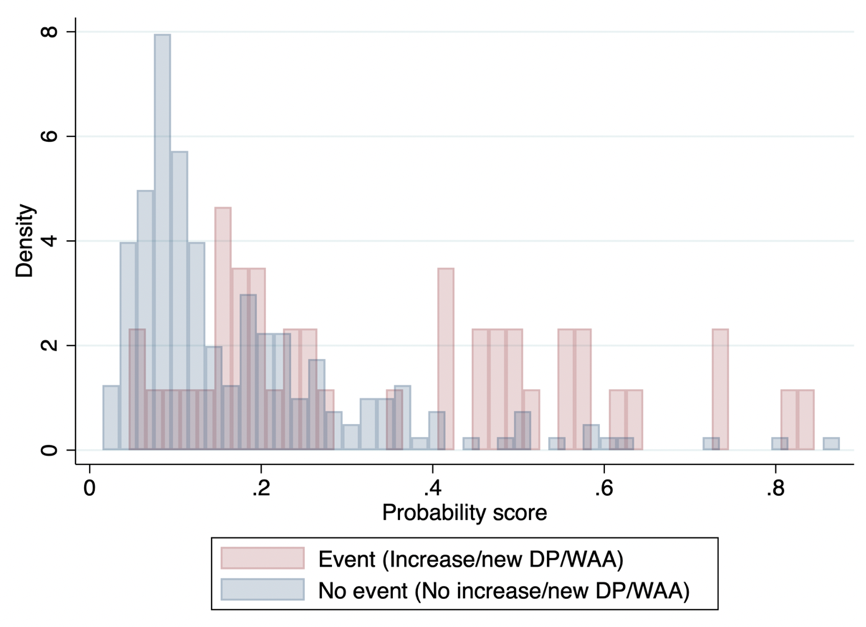 |

S3 Figure. Sensitivity analysis of complete case analyses on our external validation procedure.

| >90 days model  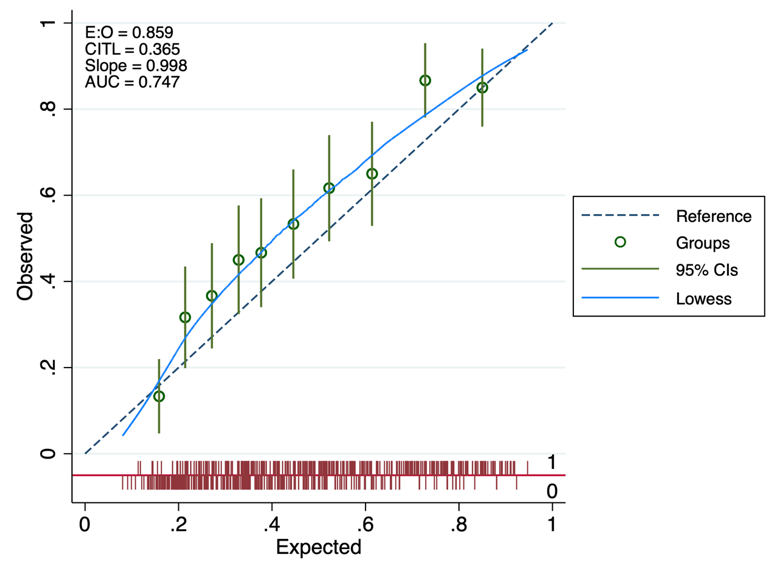 |
| --- |
| >180 days model  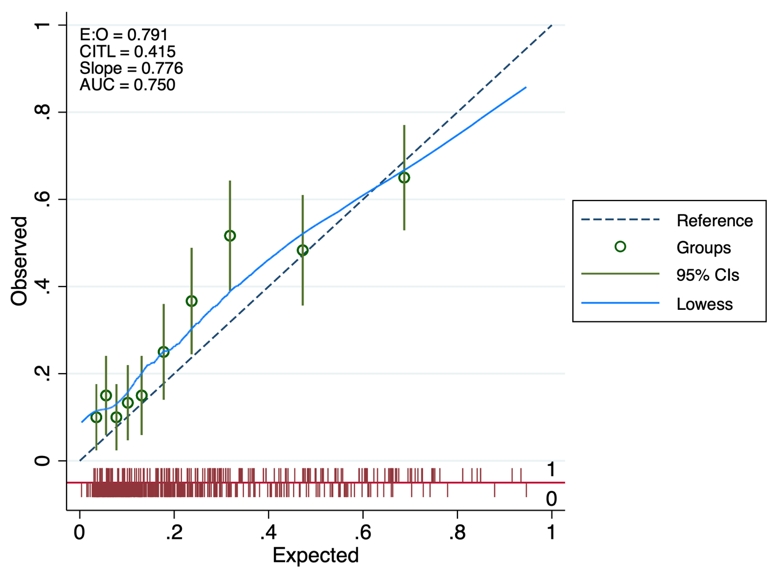 |
| DP/WAA model  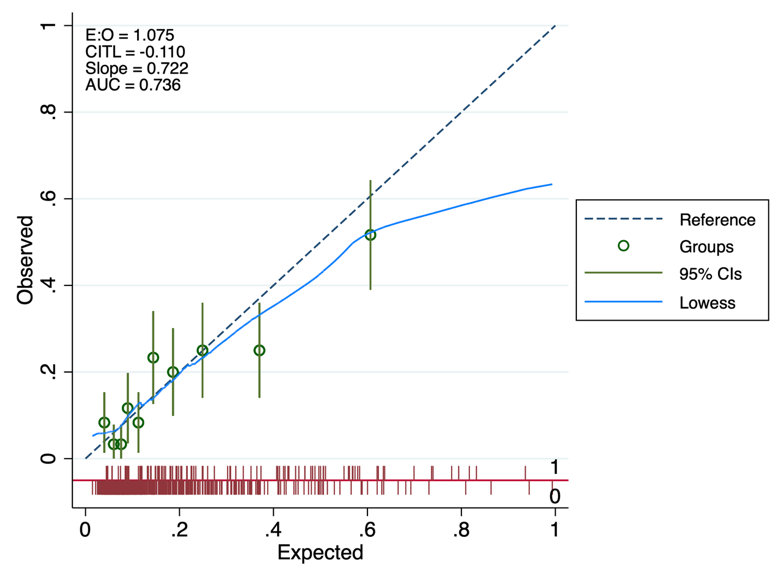 |

S4 Figure. Sensitivity analysis on all participants in the external validation cohort who were on sick leave between 4-12 weeks at baseline irrespective of diagnoses (n=600).

| >90 days model  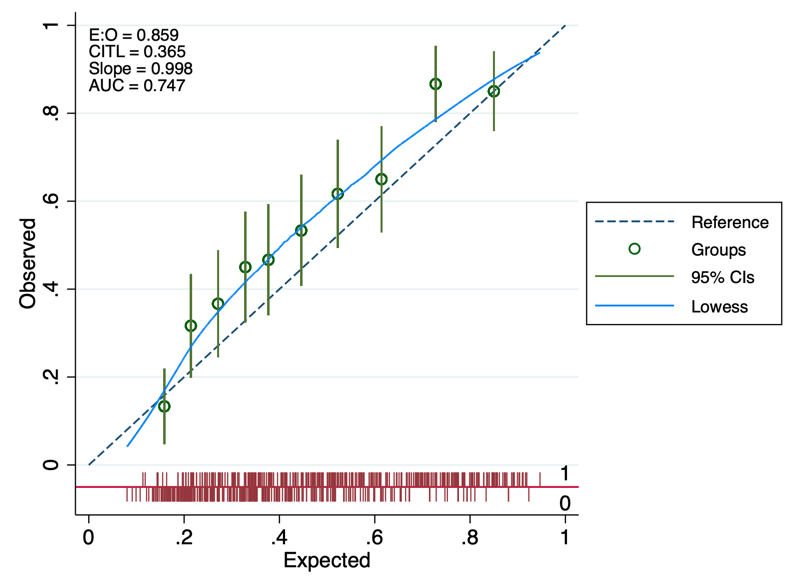 |
| --- |
| >180 days model  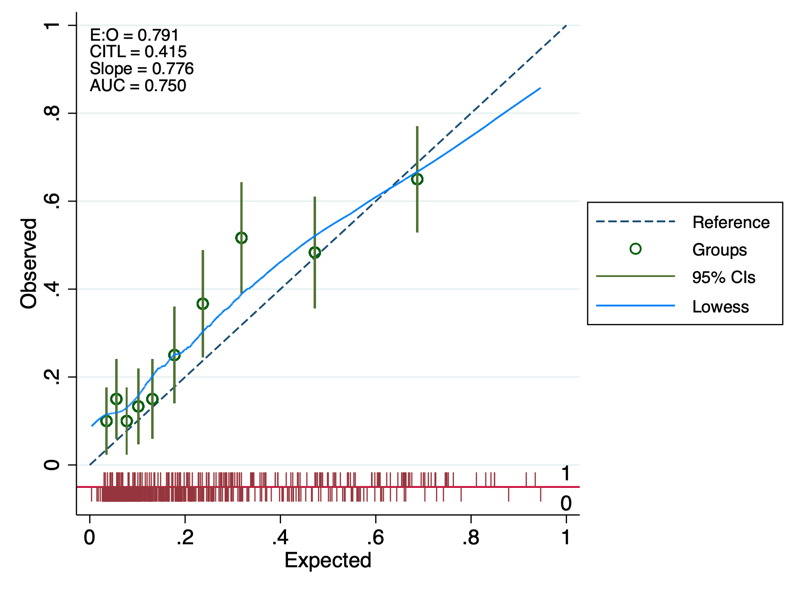 |
| DP/WAA model  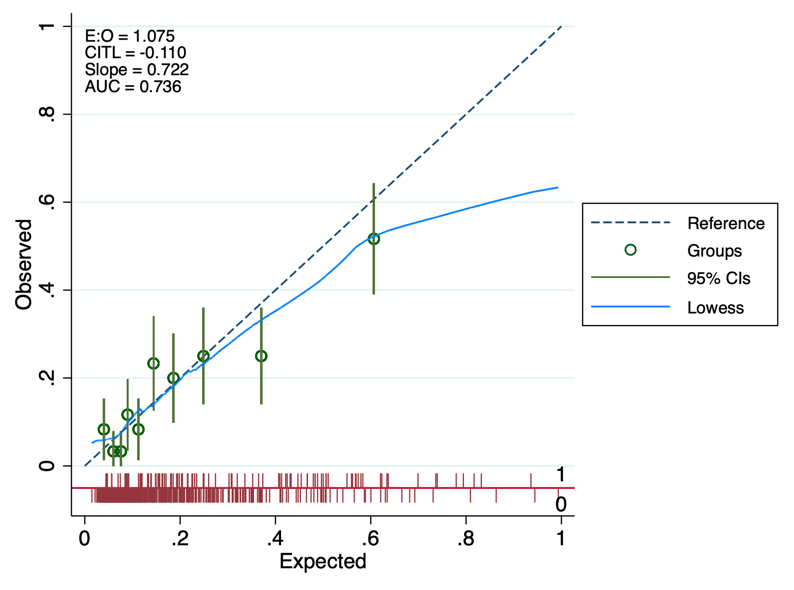 |

S5 Figure. Sensitivity analysis of the models’ performance in participants in the external validation cohort with musculoskeletal and psychological diagnoses (L and P diagnoses according to ICPC-2) (n=418).

| >90 days model  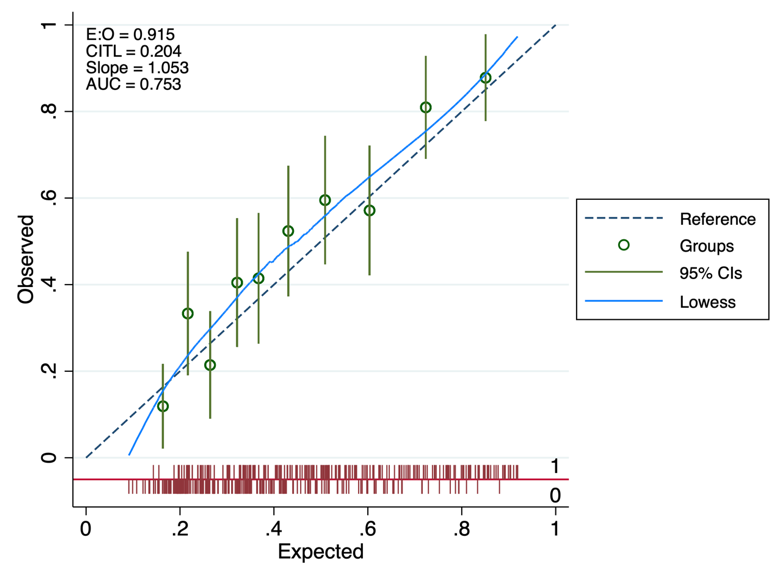 |
| --- |
| >180 days model  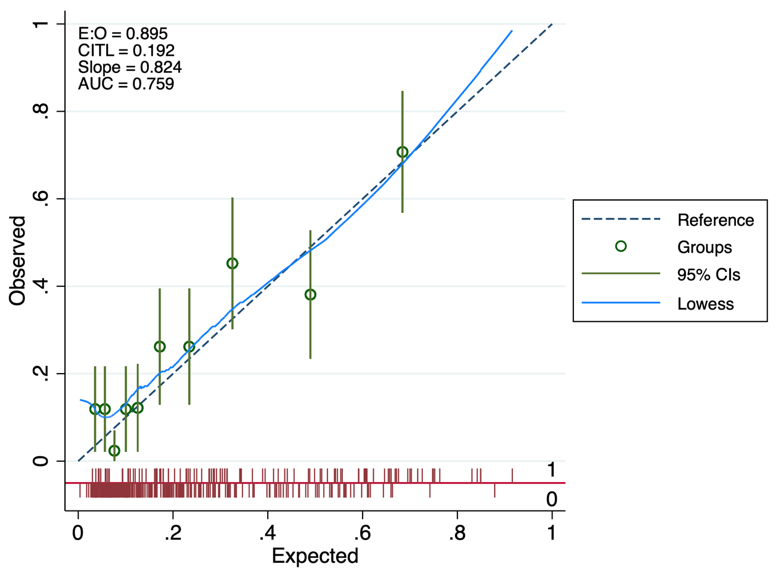 |
| DP/WAA model  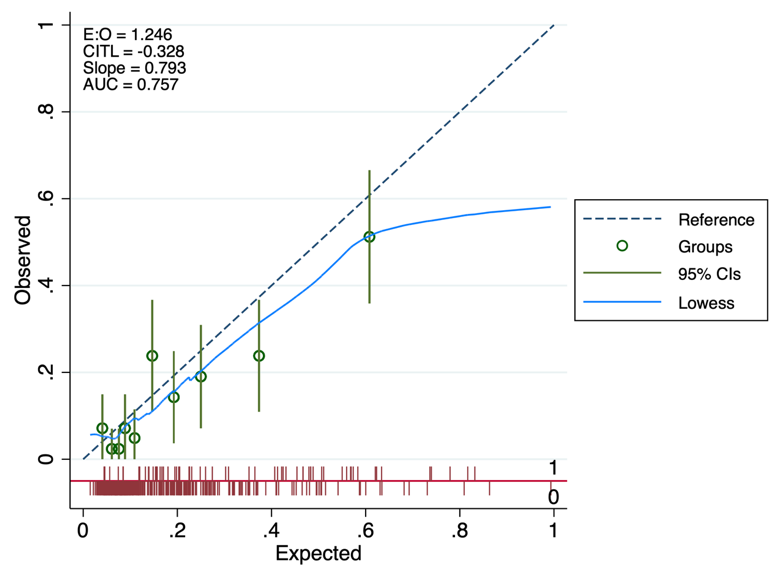 |

S1 Supporting Information: Sample size calculation

The sample size was fixed at 934 participants for the development sample. To adequately power the regression analysis, we followed recent sample size recommendations by Riley et al. [71,72] using the *pmsampsize* module in STATA 16.1 (StataCorp LLC, Texas, USA).

To include all 11 predictors and including 2 parameters for each continuous predictor to account for potential non-linear trends, 19 parameters (*P*) was needed. For sample size calculation, we used an expected shrinkage factor (*S*) of 0.9, to reflect small optimism in predictor effect estimates, as recommended by Riley et al. [72]. We assumed that the prediction models would yield modest Nagelkerke’s *R*^2^ of 25% which corresponds to a Cox-Snell R^2^ of 0.185. ﻿

**For the >90 days outcome**: The event rate was 0.403 (416 events of 934), which resulted in a minimum required sample size (*n*) of 826 (with 368 events), resulting in an event per predictor parameter (EPP) of 19.35.

**For the >180 days outcome**: The event rate was 0.172 (161 events of 934), which gave a minimum required sample size (*n*) of 826 (with 143 events) and an EPP of 7.48.

**For the WAA/DP outcome**: The event rate was 0.108 (101 events of 934), which resulted in a minimum required sample size (*n*) of 826 (with 90 events) and an EPP of 4.7.

S2 Supporting Information: Multiple imputation

**Method**

Since our missing data was assumed to be missing at random (MAR), a listwise deletion could lead to biased results. Hence, we imputed the missing data using multiple imputation. We used different approaches, such as chained and chained with predictive mean matching. We ended up running several models, from simple to more complex. For each model we checked the descriptive summaries, variance (RVI and FMI), and Monte Carlo error rules.

**Imputation model**

| **Predictors** | **Outcome** | **Auxillary variables** |
| --- | --- | --- |
| Age | >sa90 days | Kinesiophobia |
| Health | >sa180 days | Motivation RTW |
| Pain | WAA/DP | Self-efficacy RTW |
| Workability |  | Health (categorical) |
| Education |  | Pain (categorical) |
| Fear avoidance |  | Sickness absence days (cont.) |
| Disability pension rate |  | Expectation sick leave (cat.) |
| RTW expectancy |  | Fatigue |
| Sick days previous year |  |  |
|  |  |  |

**Traceplots**


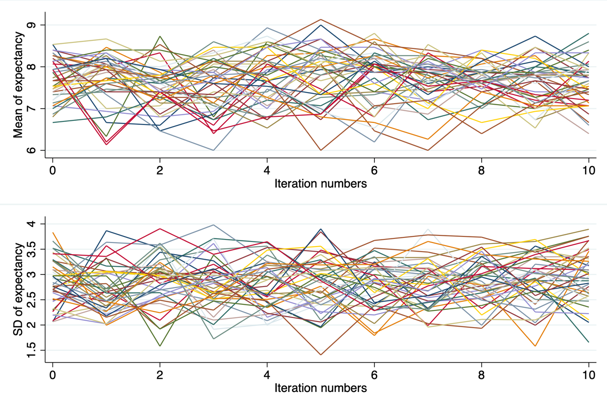

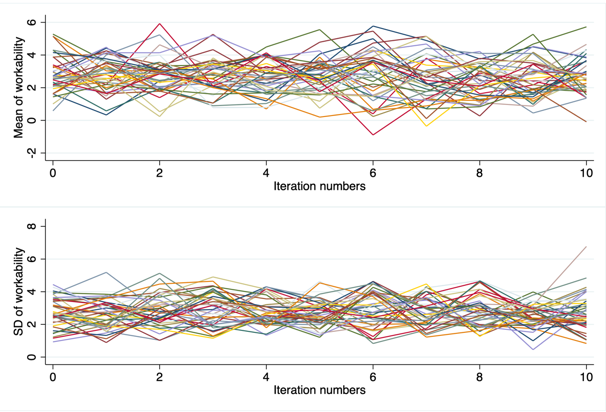


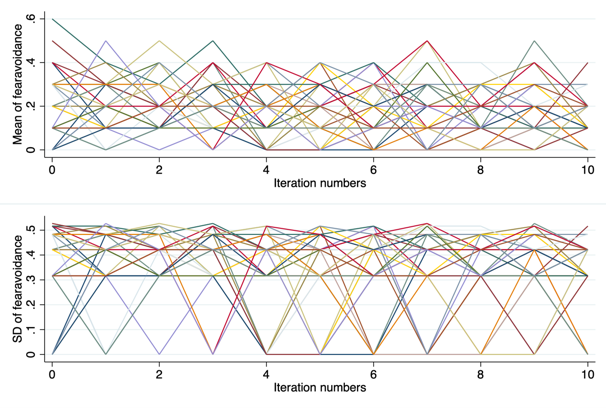

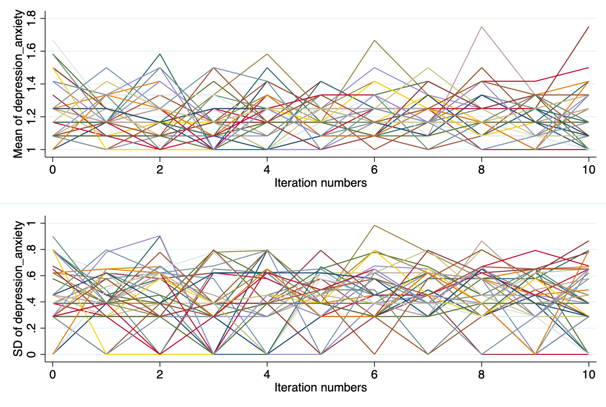


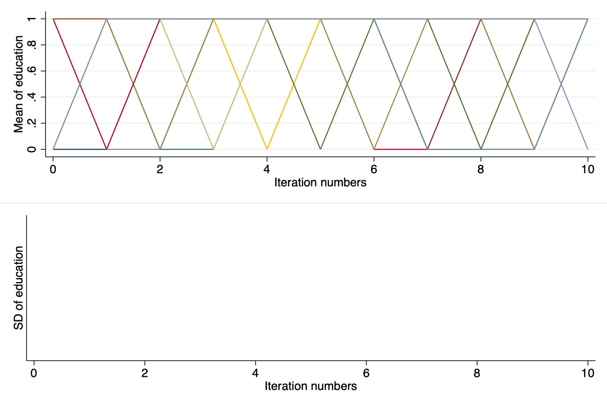

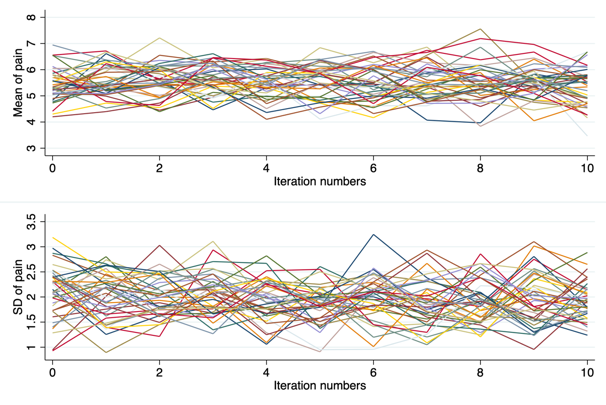


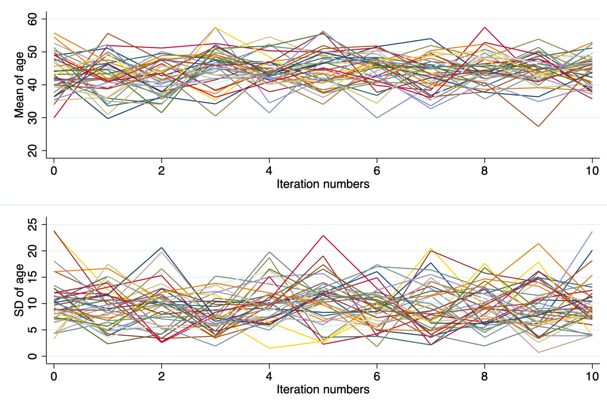

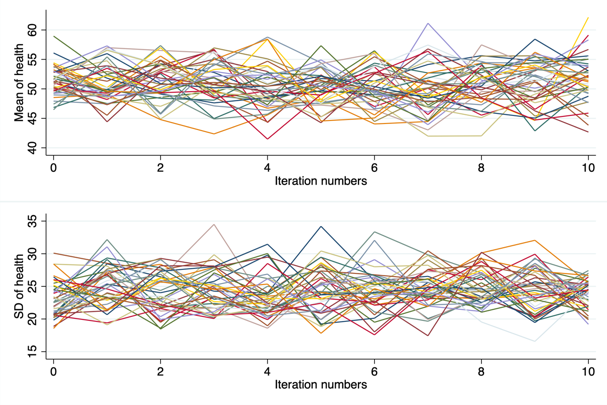


**Boxplots of continuous variables across all imputed datasets (1-40) and complete cases (0)**


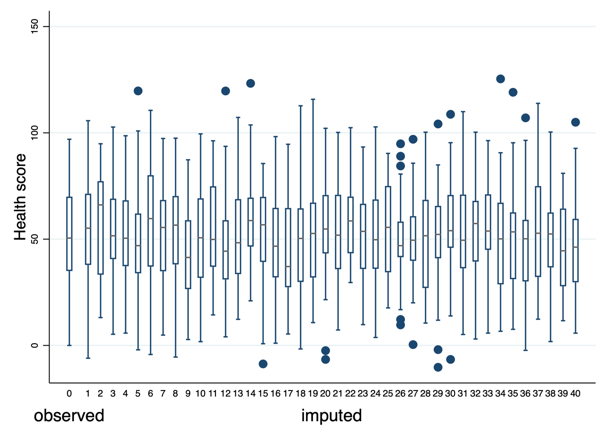

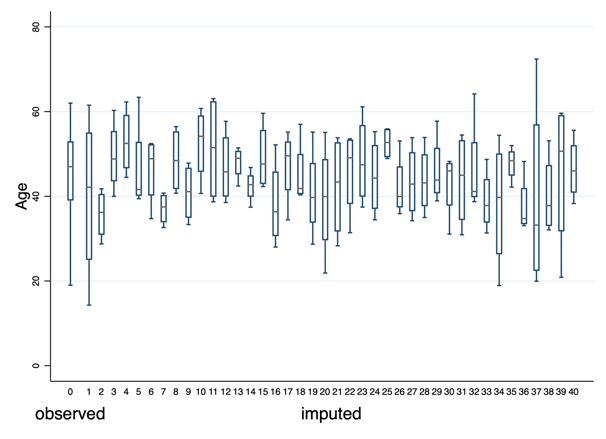


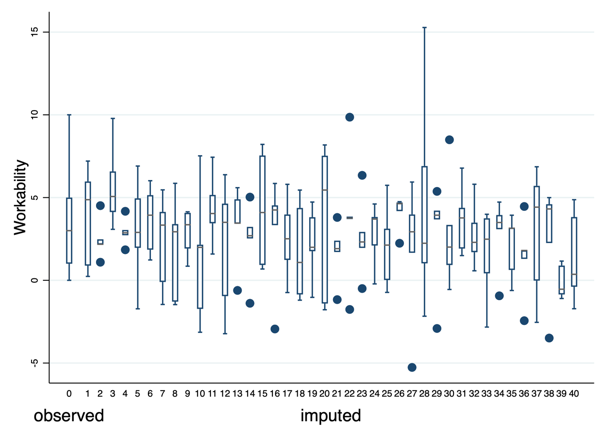

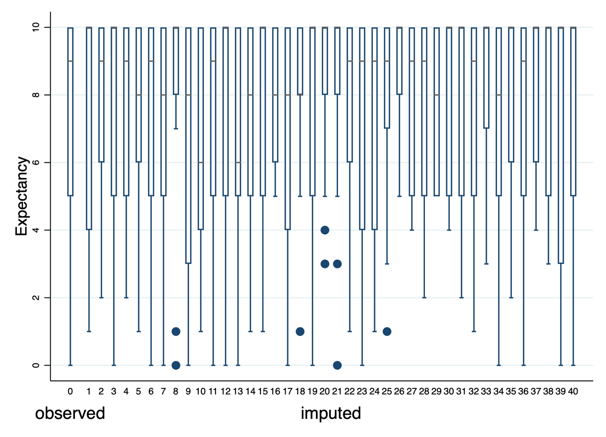


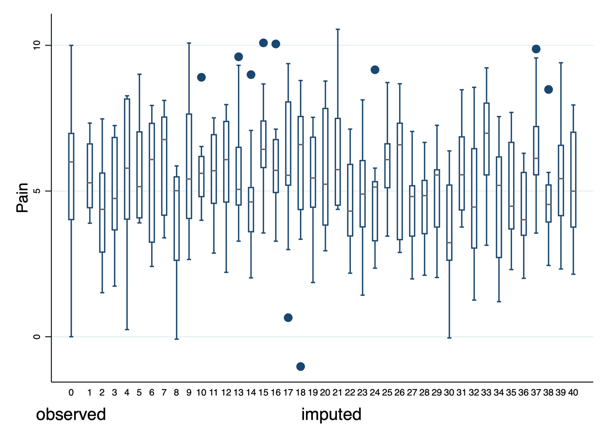


# S3 Supporting Information: Predicted Risk Calculation for >180 Days of Prolonged Work Absence with Two Examples

| **Risk score** = -0.56828409 - 0.00591762*age + 0.30552143*education + 0.08238514*pain - 0.2060209*RTW expectancy + 0.01278885*sick leave days previous year +0.00362923*health -0.43441944*[depression anxiety==2] -0.05207808*[depression anxiety==3] -0.21466543*workability -0.04010007*fear avoidance -0.06361936*disability pension status at prediction time - 0.27568155*attended an intervention/rehabilitation programme.  Age (years), pain (0-10, 10=worst), expectancy (0-10, 0=worst), sick leave days prior year, health (0-100, 0=worst), and workability (0-10, 0=worst) are coded as continuous variables. Higher education, fear avoidance, disability pension, and intervention as dichotomous (1=presence). Depression or anxiety symptoms as categorical (1=no/little, 2=moderate, 3=severe/extreme).  The predicted risk of prolonged work absence for more than 180 days can be calculated using:  ﻿exp(Risk score) / (1 + exp[Risk score]).  **Example 1**. A 40-year-old woman who was on sick leave for 51 days last year is currently on sick leave due to musculoskeletal pain. She is highly educated, does not have fear-avoidance behaviour, reports workability of 5, a health score of 56, and pain level of 5. She believes there is an 60% (6, on a scale from 0-10) chance that she will be back to work in three months. She reports little to no symptoms of depression or anxiety. She is not on disability pension and has not participated in any rehabilitation programs. She has a risk score of -1.85 and a predicted risk of 14% of being on an extended sick leave for over 180 days.  **Example 2**. A 57-year-old man, with higher education, was absent from work due to musculoskeletal pain for a total of 109 days last year. He reports a 0% chance of returning to work in 3 months and rates his overall health at 20. He does not exhibit fear-avoidance behaviour, has a work capacity of 0, reports a pain intensity of 8, and exhibits extreme symptoms of depression. He is not on disability benefits and is not participating in a rehabilitation program. He has a risk score of 1.17 and a predicted risk of 76% of being on an extended sick leave for over 180 days. |
| --- |

# References

1 Linton SJ, Nicholas M, Macdonald S. Development of a Short Form of the Örebro Musculoskeletal Pain Screening Questionnaire. *Spine (Phila Pa 1976)* 2011;**36**:1891–5. doi:10.1097/BRS.0b013e3181f8f775

2 EuroQol Group. EuroQol--a new facility for the measurement of health-related quality of life. *Health Policy* 1990;**16**:199–208. doi:10109801

3 Rysstad T, Grotle M, Aasdahl L, *et al.* Stratifying workers on sick leave due to musculoskeletal pain: translation, cross-cultural adaptation and construct validity of the Norwegian Keele STarT MSK tool. *Scand J Pain* 2022;**22**:325–35. doi:10.1515/sjpain-2021-0144

4 Dunn KM, Campbell P, Lewis M, *et al.* Refinement and validation of a tool for stratifying patients with musculoskeletal pain. *European Journal of Pain* 2021;**25**:2081–93. doi:https://doi.org/10.1002/ejp.1821

5 Lundin A, Leijon O, Vaez M, *et al.* Predictive validity of the Work Ability Index and its individual items in the general population. *Scand J Public Health* 2017;**45**:350–6. doi:10.1177/1403494817702759
